# Advanced Intelligent Control through Versatile Intelligent Portable Platforms

**DOI:** 10.3390/s20133644

**Published:** 2020-06-29

**Authors:** Luige Vladareanu

**Affiliations:** Department of Robotics and Mechatronics, Institute of Solid Mechanics of the Romanian Academy, 010141 Bucharest, Romania; luigiv2007@gmail.com

**Keywords:** intelligent control, robot control intelligent sensor systems, intelligent decision support systems, versatile intelligent portable platforms, new technologies, adaptive sensor networks, virtual and augmented reality, intelligent remote control and communication

## Abstract

Deep research and communicating new trends in the design, control and applications of the real time control of intelligent sensors systems using advanced intelligent control methods and techniques is the main purpose of this research. The innovative multi-sensor fusion techniques, integrated through the Versatile Intelligent Portable (VIP) platforms are developed, combined with computer vision, virtual and augmented reality (VR&AR) and intelligent communication, including remote control, adaptive sensor networks, human-robot (H2R) interaction systems and machine-to-machine (M2M) interfaces. Intelligent decision support systems (IDSS), including remote sensing, and their integration with DSS, GA-based DSS, fuzzy sets DSS, rough sets-based DSS, intelligent agent-assisted DSS, process mining integration into decision support, adaptive DSS, computer vision based DSS, sensory and robotic DSS, are highlighted in the field of advanced intelligent control.

## 1. Introduction

Advanced intelligent control is a rapidly developing, complex and challenging field with great practical importance and potential, which is addressed by the authors to foster the advance of science and technology and provide the theoretical and practical considerations of intelligent control techniques and their application using intelligent sensors, integrated through versatile intelligent portable platforms.

Intelligent control is the control method which imitates human intelligence in learning, decision-making and problem solving. Human characteristics consist of experience, learning, adapting and changing methods of approach and solving problems. Intelligent control techniques allow for the development of an environment which leads to recreating the advantages of natural intelligence with artificial intelligence. Advances in sensors, actuators, computation technology and communication networks help provide the necessary tools for the implementation of intelligent control hardware. Practical applications using intelligent sensors for this control method, emerged from artificial intelligence and computer-controlled systems as an interdisciplinary field, are aimed toward a variety of relevant scientific research fields on machine learning, including deep learning, bio-inspired algorithms, petri nets, recurrent neural networks, neuro-fuzzy control, bayesian control, genetic control, intelligent agents (cognitive/conscious control), extensions to traditional techniques—such as Neutrosophic logic—Extenics control, and artificial intelligence in general.

Creating new technologies using advanced intelligent control through versatile intelligent portable platforms involves complex multidisciplinary research covering: enhanced IoT technologies and applications in the 5G densification era; bio-inspired techniques in future manufacturing enterprise control; cyber-physical systems approach to cognitive enterprise; developing the IT Industry 4.0 concept; industrial systems in the digital age; cloud computing; robotics and automation with applications such as human aid mechatronics moving in unstructured and uneven environments; rescue robots; firefighting robots; rehabilitation robots; robot-assisted surgery; domestic robots. 

## 2. Review of the Contributions in This Special Issue

Advanced intelligent control is an inter-disciplinary field which combines and extends theories and methods from control theory, computer science and operations research areas with the aim of developing controllers which are highly adaptable to significant unanticipated changes. 

Illustrative wide palette approaches in advanced intelligent control through versatile intelligent portable platforms are presented in this Special Issue.

“The Intelligent Cyber Enterprise” starts from new emerging paradigms, such as the Internet of Things and Cyber-Physical Systems, focused on adopting new technologies in order to become agile, safe and productive, and capable of interoperating with smart manufacturing applications [1]. The paper introduces the concept of the intelligent cyber-enterprise using information and knowledge dynamics focusing on the importance of appropriately adapting external and internal perceptions of an enterprise through a new generation of sensorial systems—the perceptive interfaces which led to new concepts on intelligent interface instance registry, intelligent interface repository, semantic routing systems and middleware ontology, etc. 

The main characteristics of Intelligent Cyber Enterprise, in order to become agile, safe and productive, are identified: Perception; Mobility of Systems; Human–Machine Interaction; Agility of Industrial Systems; Embodied AI and Data Generation for Manufacturing; Collaboration; Safety Performance. Utilizing these characteristics allow machines interoperate with smart manufacturing applications and to better evolve. How data acquired from sensors or other system components can be analyzed and used in determining interface behaviors are demonstrated in this paper.

The Intelligent Cyber Enterprise model integrates key functions, such as processing, perception, communication, learning, pattern recognition, data mining facilitating the system adaptation to a dynamic working environment and illustrating the advantages in relation to complex system behavior modeling.

Object segmentation masks instance classification, together with YOLOv3 with improved design. 3D object reconstruction and prediction with an extended YOLOv3 network is addressed through intelligent versatile applications using full 3D, depth-based two streams, especially in the scenarios of intelligent remote control and communications, where virtual and augmented reality will soon become outdated and are forecast to be replaced by point-cloud streams, providing explorable 3D environments of communication and industrial data [2]. A hybrid artificial neural network for reconstructing polygonal meshes using a single RGB D frame and prior knowledge is proposed. The method entails the requirement of a priori information for the captured object reconstruction and a need for a large well-labelled element dataset. The training data consist exclusively of synthetically generated datasets which use ShapeNetCore, a subset of the ShapeNet dataset that provides 3D object models spanning 55 categories. 

The real-life data acquired by the Intel Realsense ZR300 and Intel Realsense D435i (Intel Corp., Santa Clara, CA, USA) devices were used for visual validation, being impossible to measure objectively without having a 3D artist recreating a 1:1 replica of said objects. The modified hybrid artificial neural networks have improved the reconstruction results by 8.53%, which allows for the much more precise filling of occluded object sides and a reduction in noise during the process.

The Special Issue continues with the presentation of six papers that deepen the scope and discuss new trends in the design, control and applications of the real-time control of robots, mechatronic systems, and human aid mechatronics or HAM (Human Adaptive Mechatronics) using advanced intelligent control methods and techniques.

In the field of the intelligent rehabilitation robots, two papers aim to help patients in achieving complete active rehabilitation training. 

New active rehabilitation training on lower limb robot motion measurement is approached, based on the dynamic modeling of human–machine coordination, using the static torque sensors for detecting the patient leg motion intention [3]. Using the dynamic relationship between the patient leg and robot leg mechanism and the variation of the leg mechanism joint torques, an innovative modeling is analyzed by which the robot completes the patient’s motion intention for active rehabilitation training. The mechanism and the hardware control system design of the LLR-Ro, the patient’s lower limb motion intention acquisition, the angular position and angular velocities of each rod centroid, each joint torque and the contact force between the patient’s leg and the leg mechanism were identified, modeled and determined. Based on the variations in the joint torques, the principle of detecting the volunteer motion intention is clear and feasible.

The patient leg motion intention and the active rehabilitation training, which improve the patient’s training initiative and accelerate the rehabilitation process, are detected based on the variation of torque sensors installed on the leg mechanism joint axis, LLR-Ro, the active training control strategy, using the joint static torque sensors and motion intension acquisition, correlated with biomechanics concepts.

An alternative to the process of rehabilitation training for stroke patients, compared to most of the methods, which process EMG signals or oxygen consumption for patients’ participation measurements, uses high cost and high complexity robotic devices, a multi-sensor system robot with torque and six-dimensional force sensors integrated in advanced intelligent control, applying the support vector machines [4]. The support vector classifiers and regression machines were used to predict the degree of the patient’s task participation, taking into account the small sample and non-linear data of the patients’ training and questionnaire data. The C and σ parameters, reported to the patients’ participation, are optimized by the hybrid quantum particle swarm optimization and support vector machines (Hybrid QPSO-SVM) algorithm. QPSO optimizes two key parameters, the C and σ of the MLSSVM, and the optimization goal is minimizing the fitness (σ, γ) function.

A further challenge is advancing intelligent control on improving upon such clinical trial data. The task difficulty can be judged and predicted online, and the assistant force adjusted in real time, for active and optimal training.

The intelligent haptic robot-glove (IHRG), for the monitoring or control of human behavior, is well described by the fractional order model (FOM) operators for the rehabilitation of patients that have a diagnosis of a cerebrovascular accident [5]. An exoskeleton architecture ensures the mechanical compliance of human fingers. The modelling is based on Lyapunov techniques, the methods that derive from Yakubovici–Kalman–Popov lemma, the frequency criteria that ensure asymptotic stability of the closed loop system and an observer control for the complex models, exoskeleton and sensors.

The use of the dynamics of an exoskeleton hand through fractional order proposes intelligent control solutions for a larger class of complex systems, such as hyper-redundant systems. An IHRG versatile intelligent portable platform, attached to the exoskeleton, supports the human hand activities by using the driving and skin sensor system, including an intelligent control for dexterous grasping and manipulation, mimicking the mechanical compliance of human fingers and determining comfortable and stable grasping functions.

Advanced intelligent control opens new approaches for high precision positioning, based on the adaptive Kalman fusion algorithm for multiple cameras and fiducial markers using multiple sensor data in complex infrastructures, in which the issue of proper information implies complex considerations with respect to system dynamics, flexibility, efficiency, safety and in defining the emergent interaction with a highly dynamic and sparsely defined environment [6]. The use of solid target alignment, in which multiple optical diagnostics are positioned using motorized 3–6 DOF manipulators, utilizes multiple instruments which need to be precisely positioned relative to each other during the experiment. The method could achieve the relative and simultaneous positioning of multiple fiducial markers in the development of advanced applications.

The Connected Bike concept combines several technologies—both hardware and software—to provide a modern alternative solution for the training and processing of training data via an internet server, leading to smart connected bike for personalized training, using the interaction of future-oriented mobility and state-of-the-art data technology [7]. Taking into account the multiple IoT specific architectures, the most suitable one for this research proved to be a hybrid open architecture, including sensors, microcontrollers, web applications, GPS module, wireless and infrared communications.

Developed as an IoT system, the Connected Bike system has a server for the management of the MQTT broker, monitoring intermediation of messages between clients, and the Back-End web application for holding the data transmitted from the bicycle and providing functionality to the Front-End. The Connected Bike system uses a wide range of technologies, starting from the electronics and hardware side, to the web and mobile applications.

A portable air scanning system was developed using a quadcopter, equipped with an air scanning sensor to perform air quality measurements. Using the Computational Fluid Dynamic (CFD) simulation, the vortex field generated by the propeller was analyzed to determine the best place for sensor mounting in order to increase the response and the accuracy of the sensor-collected data [8]. The grid pattern, with a point source and non-point source, and a wind algorithm were integrated into the gas measurement process. The DM+V kernel algorithm is used for the analysis of gas dispersion, measured by the quadcopter using convolution with a two-dimensional Gaussian kernel.

A versatile intelligent portable platform permits the quadcopter to perform optimally, reaching the target point set through the GPS coordinates. Quadcopter flight behavior, in the form of altitude, speed and measurement pattern, includes an open source web application on the ground station, which allows for remote controlling to force the quadcopter, in case of an emergency, to make a crash-landing or to fly back to the home coordinate. The flight controller is required to maintain the stability of the maneuvers and the sniffer system for performing air scanning, and also for saving data on a memory card. The analysis using normal dispersion and ANOVA were essential to obtaining increased accuracy in terms of the gas concentration and gas source position.

Among intelligent portable platforms, smartphones are some of the most ubiquitous [9]. They provide primary access to the internet and modern amenities, hold our private data and are becoming one of the primary means of attack against the user, be it through power viruses (or other means to consume resources) or more ordinary malware menaces (calling or texting tolled numbers, installing unwanted software, sending the attacker private information about the device or its owner, spying on the owner using the camera or microphone, etc.). The steps involved in obtaining a set of relevant data sources and the accompanying method using software-based sensors to detect anomalous behavior in modern smartphones, based on machine-learning classifiers, are described. 

The purpose of this study was to assess if anomalous behavior could be detected through machine-learning classifiers, based on input data sources from a variety of sensors within the device. Three types of classifiers for the machine-learning application—logistic regression, a shallow neural network for pattern recognition, and SVMs—are investigated. The three are evaluated on several metrics, the most important of which being the F1 score on the test set. The full details of the design, implementation, and evaluation of the learning algorithms are presented step by step in their respective sections. 

The results show that all the three investigated algorithms perform reasonably well, with SVMs having a slight edge. The dataset split procedure, discussed at length throughout the paper, give the model a good ability to generalize as yet unseen data.

The effectiveness of several deep learning models of facial expression recognition, such as SSD and fast R-CNN for human–robot interaction is very interesting, deeply analysed and verified [10]. Based on an innovative end-to-end pipeline method that applies two optimized CNNs, the face recognition (FR) and the facial expression recognition (FER), using deep convolutional neural networks (CNN) for the growth real-time inference speed of the entire process is achieved, leading to a high level of advanced intelligent control in the interaction between humans and a NAO robot.

The paper focuses on enhancing the performances of different types of convolutional neural networks (CNN), in terms of accuracy, generalization and inference speed, using several optimization methods (including rectified Adam), such as FER2013 database augmentation with images from other databases and asynchronous threading at inference time. For emotion recognition, transfer learning and the fine-tuning of three CNN models (VGG, Inception V3 and ResNet) have been used. The outcomes prove improvements over 10% when using two serialized CNN, instead of when using only the FER CNN, while the recent optimization model, called rectified adaptive moment optimization (RAdam), lead to a better generalization and accuracy improvement of 3–4% on each emotion recognition CNN. The innovative end-to-end pipeline uses deep convolutional neural networks for training real-time accurate models, which can be applied to human–machine interactions on humanoids robots or other intelligent portable platforms in order to obtain advanced intelligent control.

Advanced intelligent control in micro-nano technologies is addressed in the following papers.

The development of sensors that will lead to new trends in real-time intelligent sensor systems through advanced intelligent control methods and techniques can be achieved through an innovative approach in the growing of nanowires on silicon for ultraviolet photodetectors [11]. The effect of silver catalysts to enhance the growing of Ga_2_O_3_ nanowires and the sensitivity of β-Ga_2_O_3_ nanowires for UV detection were investigated. Semiconductor nanowires exhibit improved material properties compared to thin-film semiconductors, becoming an ideal candidate for visible-blind UV-light sensors, such as power electronics, solar-blind UV detectors and devices for harsh environments. 

The results led to highly-oriented, dense and long Ga_2_O_3_ nanowires that can be grown directly onto the surface of silicon, forming a pn heterojunction with rectifying characteristics and excellent UV photo-response. 

The advanced intelligent control methods and techniques, which could lead to new concepts and designs in the development of the real-time intelligent sensory control systems, is approached in a novel PDMS-based sensor system for MPWM measurements of picoliter volumes in microfluidic devices [12]. An automatic microinjection system, by integrating a sensor based on image processing of the fluid that flows through microchannels using the microwire molding technique realized with the technique known as microwire-molding, was designing and achieved, validating the concept of the sensor that measures fluid volumes at picolitric levels or lower. The microfluidic devices have wide applications in biological and medical analysis and in the detection, control and manipulation of biological samples and cell biology research, such as in the analysis of unpurified blood samples, analysis of complex mixtures and molecules (especially DNA and proteins), DNA sequencing, single cell manipulation, electrophoretic separations, drug screening, screens for protein crystallization conditions, cell culture studies and reproductive cell selection.

A group of two papers focuses on advanced intelligent control in human health monitoring.

In total, 94 studies, summarize the state of research into the use of technology with a focus on teaching people with Asperger’s syndrome, taking into account the 13 aspects of user experience, usability and accessibility. An in-depth review shows how the use of technology in 12 educational contexts helps people with autism spectrum disorder (ASD) to develop several skills [13].

The research methodology was based on systematic literature review using the Kitchenham’ process: planning, conducting and publicizing in terms of Research Questions, Data Sources and Search Strategies, Search Strings, etc. 

The use of technological advancements such as virtual agents, artificial intelligence, virtual reality and augmented reality, allows for an environment of comfort and constant learning for people with Asperger’s syndrome.

Smart offices, enhanced and developed through assistive technologies using Brain Computer Interface (BCI) for the adaptive control of the lighting and temperature in working spaces, sensing the environment’s temperature and lighting, can respond of the user’s comfort needs [14]. The BCI acquires alpha, beta and theta powers extracted from the EEG signals, representing the worker’s comfort level. Advanced intelligent control systems, considering many factors which influence EEG, such as emotional state, fatigue, sleepiness, age, body temperature, and blood oxygen saturation, will be able to lead to the best comfort and engagement environment using artificial intelligence techniques.

A flexible automatic distortion rectification methodology, for the automatic distortion rectification of wide-angle and fisheye lens camera models, with a comprehensive mathematical model that can refine the outliers simultaneously, optimizing the best-fit parameters with minimum error possible, is proposed [15]. An iterative optimization was used with the refinement of the outliers from the pool of robust line-member set, the identification of the plumbline angular cumulative loss over refined line-member set and an investigation into the significance through an ablation approach. The system is congruent quantitative vs. accuracy, practical significance and qualitative vs. adaptability and processing time, in relation to the real/synthetic, public and private datasets.

Relevant experiments for image quality, stretching and pixel-point error on various metrics, such as PSNR, SSIM, S3 and LPC-SI, with greater precision regarding distortion compensation and maintaining pixel consistency in the context of employing wide-angle lens models for applications on advanced driver-assistance system (ADAS) and video surveillance, were extensively exploited to validate the automatic distortion rectification of wide-angle images.
